# 38-year-old Woman with a Cough and a Rash

**DOI:** 10.5811/cpcem.2018.11.40591

**Published:** 2019-01-04

**Authors:** Megan P. Donohue, Elizabeth P. Clayborne, Zachary D.W. Dezman, Laura J. Bontempo

**Affiliations:** * University of Maryland Medical Center, Department of Emergency Medicine, Baltimore, Maryland; † University of Maryland School of Medicine, Department of Emergency Medicine, Baltimore, Maryland

## CASE PRESENTATION (Dr. Donohue)

A 38-year-old female presented to the emergency department (ED) with rash, dyspnea, odynophagia, and nasal congestion for the prior two weeks. During that time, she sought medical care twice. The first physician to evaluate the patient started her on antibiotics for a presumed upper respiratory infection (URI). Her symptoms did not improve after completing a 10-day course of amoxicillin; then a second medical provider prescribed her ciprofloxacin. She was on her eighth day of ciprofloxacin (i.e., total 18^th^ day of treatment) when she presented to our ED with rash and dyspnea. She decided to come to the ED because her cough had worsened and become productive of sputum. She also complained of one month of fevers, chills, night sweats, and malaise. She denied any complaints of headaches, chest pain, palpitations, abdominal pain, genitourinary or neurologic symptoms.

Her past medical history was significant for adult-onset asthma and allergic rhinitis. Medications included fluticasone, ipratropium, and her recent courses of amoxicillin and ciprofloxacin. She had no known medication allergies but reported gastrointestinal intolerance to fish oil. Her family history was significant for a sister with multiple sclerosis. She was an Iranian immigrant who had moved to Baltimore six months prior to presenting in our ED. She was married with no children and denied ever using tobacco, alcohol or illicit drugs.

On physical exam, she was alert but appeared uncomfortable as she hobbled into triage that night. She was afebrile (36.7° Celsius) and mildly tachycardic (heart rate of 110 beats per minute). Her blood pressure was 102/68 millimeters of mercury, she was mildly tachypneic with a respiratory rate of 20 breaths per minute, and her oxygen saturation was 97% while breathing room air. She was well developed and well nourished, with an estimated body mass index of 22. Her head was normocephalic and atraumatic. Her oropharynx was clear; her neck was supple and no lymphadenopathy was detected. On auscultation, she was tachycardic with a normal S1 and S2, without any murmurs, gallops or rubs. She was mildly tachypneic without any accessory muscle use, retractions, or increased work of breathing. She was able to speak in full sentences without difficulty. The patient’s lungs were clear to auscultation bilaterally without wheezes, rhonchi, or rales. Her abdomen was soft and non-tender, and no lower extremity edema was present. She was alert, oriented and appropriately interactive.

**Image 1:**
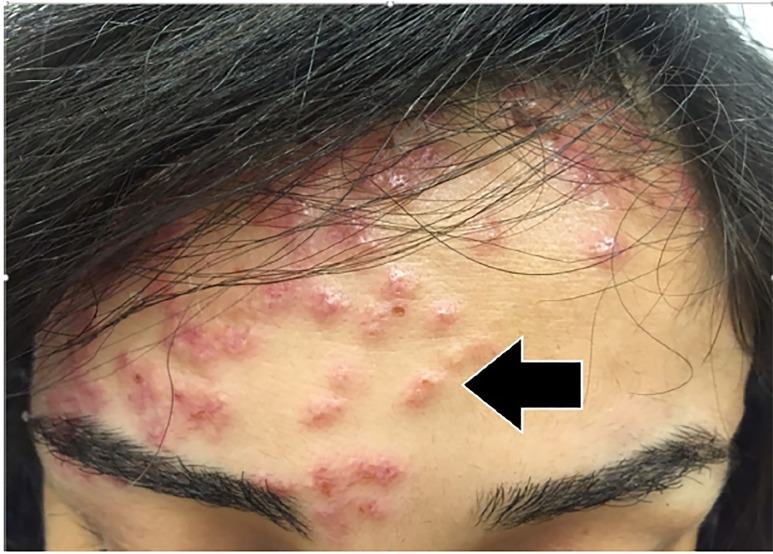
Papulovesicular eruptions with petechiae on the forehead of the patient (arrow).

**Image 2:**
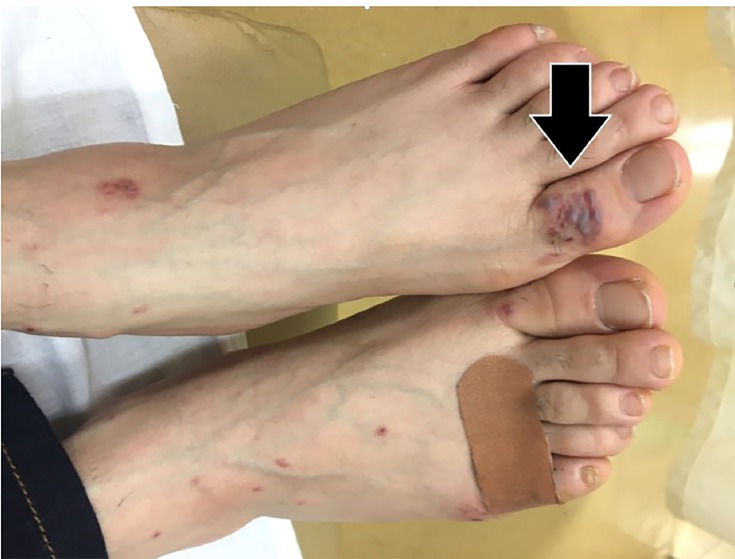
Scattered hemorrhagic vesicles (arrow) with purpuric macules on the patient’s feet.

On closer examination of the patient’s skin, her rash appeared to have three different morphologies. The first was located on her forehead and consisted of sub-centimeter papulovesicular eruptions with petechiae that were pruritic but not tender (Image 1). The second rash consisted of scattered hemorrhagic vesicles with purpuric macules and was located on her distal upper and lower extremities (Image 2). The third rash was an erythematous and indurated plaque at the base of the left foot, which was tender and made it painful for her to walk.

**Image 3:**
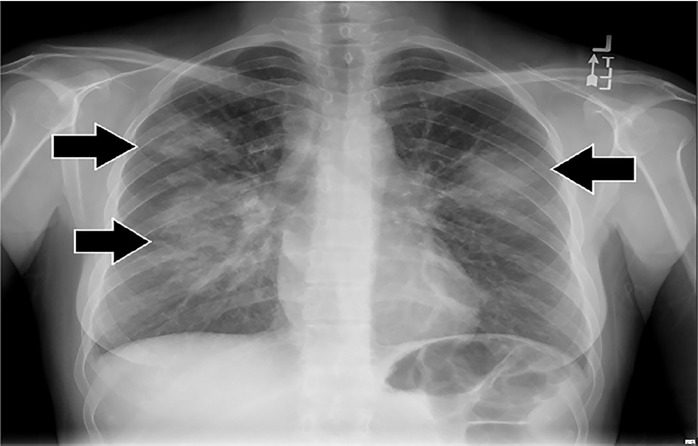
Chest radiograph of a 38-year-old patient with rash and dyspnea, showing bilateral multi-lobar infiltrates (arrows).

Initial laboratory results are shown in Tables 1–3. The patient’s electrocardiogram showed sinus tachycardia with normal intervals and without ST-segment or T-wave abnormalities. Bilateral multilobar infiltrates were revealed on chest radiograph (Image 3). A computed tomography (CT) of her chest confirmed the presence of bilateral multilobar infiltrates and a CT of her sinuses showed mucosal thickening throughout. An echocardiogram revealed a normal ejection fraction and no valvular pathology. No vegetations were seen. The patient was admitted to the hospital for further evaluation. A test then was performed, which confirmed the diagnosis.

## CASE DISCUSSION (Dr. Clayborne)

My approach to cases that contain a lot of non-specific information is to first look at the big picture. I like to identify highlights from the history of present illness (HPI), past medical history, social history, review of symptoms (ROS) and physical examination to isolate what stands out most and may give insight into the differential. First impressions of the HPI were the story of a young female with a history of asthma and allergic rhinitis, who recently had a URI that was unresponsive to two different antibiotics. That patient then presented to the ED with a rash after taking ciprofloxacin. First impressions of the ROS highlighted that she had one month of constitutional symptoms and fatigue, nasal congestion, sore throat, a cough that was productive without hemoptysis and a new rash with pruritis. First impressions of social history included her status as an Iranian immigrant who did not drink, smoke or use drugs. Finally, first impressions from her physical examination included an afebrile, stable-appearing patient with tachycardia and tachypnea whose lungs were clear to auscultation bilaterally, who also had rashes of three different morphologies on her face, extremities, and the sole of one foot.

Immediately after my first impressions, the item that raised my concern the most was the rash. I find that many emergency physicians can be uncomfortable with rashes since we often find them difficult to properly describe, breaking the link between identifying a rash and making the diagnosis. In this case, the rash’s location and characteristics were not specific to an etiology with which I was familiar. But I was able to combine the description of the rash with my first impressions to generate a preliminary differential diagnosis.

I subdivided my differential diagnosis into the three broad areas: infectious (bacterial, viral, or fungal); allergic; and autoimmune. Based on these three categories I began to use the data collected in the ED to help narrow my focus.

Pertinent positives from the lab work included a mild leukocytosis of 12.1 kilo/microliter with eosinophilia of 10.6%, urinalysis with trace blood, erythrocyte sedimentation rate of 70 millimeters per hour, a C-reactive protein of 3.3 milligrams per liter, and her screen for acquired human immunodeficiency virus was nonreactive. These are the labs that I would expect to result during the patient’s ED visit. In the ED I would also see her chest radiograph and CT chest showing bilateral multilobar infiltrates. With this information, if I were the treating physician, I would order antibiotics and admit the patient for an inpatient workup.

The results that would more likely be done during the inpatient stay demonstrated sinusitis, a normal cardiac ejection fraction, and an elevated Immunoglobulin E (IgE) of 6,266 kilounits per liter. Based on this additional data, I went back to my broad differential diagnosis to see what fit and did not fit each category. Allergic etiologies would include a rash, respiratory symptoms, and perhaps mild elevation of inflammatory markers but do not account for the constitutional symptoms or positive findings on radiograph and CT. Autoimmune etiologies would account for the constitutional symptoms, rash, and infiltrates but I would question why only a few of the inflammatory markers were elevated. Infectious etiologies, especially fungal infections, could account for the constitutional symptoms, pulmonary disease, eosinophilia, and elevated IgE.

My top two diagnoses based on the patient’s presentation coupled with her laboratory results are eosinophilic granulomatous polyangiitis (EGPA), also known as Churg-Strauss syndrome, and aspergillosis. Invasive pulmonary aspergillosis starts when the patient becomes colonized by inhaling fungal spores. The patient’s symptoms are often constitutional (weakness, fatigue, and low-grade fevers) and non-specific (shortness of breath and cough that is productive but not responsive to antibiotics). Patients often present with hemoptysis, which did not occur in this case. The infection can spread from the lower respiratory tree to multiple organs, most often the brain. These patients will then develop abnormalities on head CT (infarcts, ring-enhancing lesions, hemorrhage, abscess) and begin to suffer from seizures.

Patients with aspergillosis will often present with eosinophilia, elevated IgE levels, abnormal chest radiographs and CTs, sinusitis, and a characteristic rash. The rash begins as either solitary or multiple erythematous or violaceous, indurated papules or plaques. It can be tender and evolve rapidly into pustules, hemorrhagic vesicles, or eschars.^1^ However, this patient does not have any of the risk factors for aspergillosis (prolonged neutropenia, transplant [especially lung], prolonged high-dose corticosteroid therapy, hematological malignancy, cytotoxic therapy, or advanced acquired immune deficiency syndrome. This patient has sinusitis and no hemoptysis, both of which are inconsistent with aspergillosis.

EGPA was first described in 1951 by Churg and Strauss. They described a syndrome in 13 patients who had asthma, eosinophilia, granulomatous inflammation, necrotizing systemic vasculitis, and necrotizing glomerulonephritis. In 1990 the American College of Rheumatology (ACR) proposed the following six criteria for the diagnosis of EGPA:^2^

- Asthma (wheezing, expiratory rhonchi)
- Eosinophilia of more than 10% in peripheral blood
- Paranasal sinusitis
- Pulmonary infiltrates (may be transient)
- Histological proof of vasculitis with extravascular eosinophils
- Mononeuritis multiplex or polyneuropathy

This patient met four of these criteria (asthma, eosinophilia, sinusitis and pulmonary infiltrates), which is consistent with a diagnosis of EGPA (sensitivity of 85%, specificity of 99.7%).

If I were the treating physician, I would empirically treat with antifungals due to the concern for aspergillosis. For this case exercise, however, I believe a punch biopsy would confirm a diagnosis of EGPA.

## CASE OUTCOME (Dr. Donohue)

The diagnostic study performed was a punch biopsy of a hemorrhagic vesicle on the right foot. It revealed two key findings: 1) eosinophils surrounding a central granuloma; and 2) the cross-section of a blood vessel with central necrosis. These findings confirmed the diagnosis of EGPA, also known as Churg-Strauss syndrome.

Our patient was admitted to an inpatient medical service, but the diagnostic punch biopsy was actually performed in the ED prior to admission. While on the inpatient service, numerous consultants participated in her care including dermatology, rheumatology, pulmonology, and infectious disease. She underwent bronchoscopy, broncho-alveolar lavage and lung biopsy. Ultimately, based on the ACR EGPA criteria, she was diagnosed with EGPA and started on high-dose prednisone 60 milligrams (mg) daily.^2^ On outpatient follow-up, she was transitioned to azothioprine and at one year follow-up was doing well.

## RESIDENT DISCUSSION

EGPA is a rare vasculitis with an estimated incidence of one to three cases per million people. Early in the history of its recognition, reliable data on incidence were unavailable due to its similarities with other heterogeneous disease processes and lack of diagnostic criteria.^3^ EGPA was first described in 1951 by two young pathologists in New York City who were studying vasculitis. As pathologists, their case study data was obtained from autopsies in which they recognized “the occurrence of a clinical syndrome of severe asthma, fever and hypereosinophilia.”^4^ Keep in mind, this was in addition to the fact that these patients already had known vasculitis and were already deceased. However, it was “the finding of granulomatous lesions, both within vessel walls and in connective tissue” that made it distinct from other allergic syndromes and vasculitides.^5^ Churg and Strauss theorized that 11 of the 13 sentinel cases had died due to this syndrome to which they applied their eponym.

Since 1951 the underlying pathology and pathogenesis of EGPA has become better understood. As a result, the name was changed from Churg-Strauss syndrome to EGPA, which better describes the underlying disease process and the phases of its clinical manifestations. EGPA has three distinct clinical phases. The first, or prodromic, is characterized by onset of asthma in the second or third decade of life. It often is associated with allergic rhinitis and recurrent sinusitis. The second, eosinophilic, phase is marked by peripheral eosinophilia with organ infiltration. Peripheral eosinophilia may be masked by steroid therapy.^5^ Eosinophilic organ infiltration occurs most commonly in the lungs, peripheral nerves and skin, but can occur in any organ system,^6^ creating an array of clinical presentations.^7^ This phase of the disease is difficult to distinguish from other hypereosinophilic conditions. The third phase of EGPA is vasculitic; this phase, which is unique to EGPA, makes it fatal if untreated.

Patients with pulmonary EGPA can develop pulmonary infarcts, nodules or diffuse alveolar hemorrhage. The most common CT findings include bilateral ground-glass opacities, airspace consolidation, centrilobular nodules and bronchial wall thickening.^8^,^9^ Infiltration of the heart can cause myocardial infarction, pericarditis or congestive heart failure. Central nervous system involvement may result in neuropathy, mononeuritis, seizure, stroke or coma. One study found that mononeuritis was the second most common presentation of EGPA, second only to asthma.^8^ In the gastrointestinal system EPGA can cause cholecystitis, pancreatitis or gastroenteritis. Vasculitic involvement of the renal system may result in proteinuria, hematuria, glomerulonephritis or renal insufficiency. Any of these complications may be a patient’s presenting symptoms; the case we highlighted is the most common presentation of EGPA. Organ system involvement is important due to its prognostic value as calculated by the five-factor score. If two or more of the following organ systems are involved, cardiac, gastrointestinal, nervous system or kidneys, five-year mortality is 50% untreated.^9^

Diagnostic criteria have changed over time. Churg and Strauss initially described a disease that only was diagnosed on biopsy. It was felt that an emphasis on biopsy and pathological findings led to the disease being underdiagnosed.^10^ The ACR revised the diagnostic criteria in 1990 to include more common features: the presence of asthma; eosinophilia >10% on white blood cell count differential; mononeuropathy or polyneuropathy; non-fixed pulmonary infiltrates on imaging; parasinus abnormality; or a characteristic biopsy. If four or more criteria were present, sensitivity was 85% and specificity 99.7%. Alternatively, if a patient had asthma, eosinophilia and history of allergy or drug sensitivity, sensitivity was 95% and specificity 99.2%.^2^ Now that biopsy is no longer needed to confirm the diagnosis, a provider’s clinical index of suspicion portends significant impact in recognition and diagnosis of EGPA.

It has been suggested that anti-leukotriene medications may play a role in the development of EGPA, but this is controversial.^11^–^14^ The current understanding is that prolonged survival of eosinophils due to inhibition of CD95-mediated apoptosis plays a role in EGPA pathogenesis. Recent data suggest that cytokine release from T-lymphocyte may be an important step.^15^ Even though the exact cause of EGPA has yet to be fully elucidated, treatment guidelines are available. High-dose glucocorticoids (1mg/kg/day prednisone) for at least two to three weeks is the cornerstone for treatment to obtain remission. EGPA responds well to first-line treatments, but relapse has been shown to occur in up to 25% of patients.^10^ Cyclophosphamide is the main pharmacotherapy for remission induction. Azothioprine and methotrexate are used for maintenance therapy for patients with life- or organ-threatening disease involvement. Intravenous immunoglobulin is considered second-line treatment for refractory disease. New treatment modalities currently being studied include plasma exchange and use of monoclonal antibodies.^16^ Treatment of EGPA should always include a multidisciplinary team, including rheumatology.

## FINAL DIAGNOSIS

Eosinophilic granulomatous polyangiitis, also known as Churg-Strauss syndrome

## KEY TEACHING POINTS

1. EGPA is a rare but deadly vasculitis.
2. Typical features of EGPA are asthma with allergic rhinitis and recurrent sinusitis, peripheral eosinophilia and vasculitis.
3. High clinical suspicion is paramount as it is a clinical diagnosis.
4. Differential diagnosis should be broadened when a patient has bounced back and already failed initial medical therapy.
5. Diagnostic momentum in the ED can play a pivotal role in making the correct diagnosis.

## Figures and Tables

**Table 1:** Initial laboratory results of a 38-year-old female patient presenting with a rash and dyspnea.

	Values
Complete blood cell count	
White blood cell count (K/μL)	12.1
Hemoglobin (g/dL)	11.5
Hematocrit (%)	33
Platelets (K/μL)	237
Differential	
Granulocytes (%)	64.4
Lymphocytes (%)	15.3
Monocytes (%)	8.3
Eosinophils (%)	10.6
Serum chemistries	
Sodium (mmol/L)	141
Potassium (mmol/L)	3.5
Chloride (mmol/L)	100
Bicarbonate (mmol/L)	26
Blood urea nitrogen (mg/dL)	6
Creatinine (mg/dL)	0.54
Total protein (g/dL)	8.4
Albumin (g/dL)	3.8
Aspartate aminotransferase (u/L)	26
Alanine aminotransferase (u/L)	21
Alkaline phosphatase (u/L)	58

*K*, kilo; *μL*, microliter; *g*, grams, *dL*, deciliter; *mmol*, millimoles; *L*, liter; *mg*, milligrams; *u*, units.

**Table 2:** Additional laboratory results of 38-year-old patient presenting with a rash and dyspnea.

Additional labs	Values
Human immunodeficiency virus screen	Non-reactive
Erythrocyte sedimentation rate (mm/hr)	70
C-reactive protein (mg/L)	3.3
Immunoglobulin E (KU/L)	6,266

*Mm*, millimeters; *hr*, hour; *mg*, milligrams; *L*, liter; *KU*, kilounits.

**Table 3:** Urinalysis of 38-year-old patient presenting with a rash and dyspnea.

Urinalysis	Values
pH	7
Specific gravity	1.003
Glucose	Negative
Ketones	Negative
Protein	Negative
Nitrile	Negative
Leukocyte esterase	Negative
White blood cells	None
Red blood cells	Trace
